# Predicting 30-day unplanned readmissions in patients with cancer in a Brazilian hospital: a Machine Learning approach

**DOI:** 10.31744/einstein_journal/2026AO2210

**Published:** 2026-09-02

**Authors:** Nickolas Stabellini, Marilia Melo Favalesso, Claudia Szlejf, Cesar Gomes Miguel, Roberto Carmagnani Pestana, Hanna Kim, Gabriela de Carvalho Ferreira, Kristin A. Waite, Alberto J. Montero, Jill S. Barnholtz-Sloan, Edson Amaro, Nelson Hamerschlak

**Affiliations:** 1 Case Western Reserve University Case Western Reserve University School of Medicine Cleveland OH United States Case Western Reserve University School of Medicine, Case Western Reserve University, Cleveland, OH, United States.; 2 University Hospitals Seidman Cancer Center Department of Hematology-Oncology Cleveland OH United States Department of Hematology-Oncology, University Hospitals Seidman Cancer Center, Cleveland, OH, United States.; 3 Hospital Israelita Albert Einstein Faculdade Israelita de Ciências da Saúde Albert Einstein São Paulo SP Brazil Faculdade Israelita de Ciências da Saúde Albert Einstein, Hospital Israelita Albert Einstein, São Paulo, SP, Brazil.; 4 Medical College of Georgia at Augusta University Division of Cardiology Department of Medicine Augusta GA United States Department of Medicine, Division of Cardiology, Medical College of Georgia at Augusta University, Augusta, GA, United States.; 5 Hospital Israelita Albert Einstein São Paulo SP Brazil Hospital Israelita Albert Einstein, São Paulo, SP, Brazil.; 6 Emory University Winship Cancer Institute Atlanta GA United States Winship Cancer Institute, Emory University, Atlanta, GA, United States.

**Keywords:** Neoplasms, Patient readmission, Unplanned, Machine learning

## Abstract

**Objective:**

This study aims to develop Machine Learning models to predict 30-day unplanned readmissions in cancer patients treated at a private hospital in Brazil.

**Methods:**

This retrospective cohort study included admission records of hospitalizations lasting >24 hours, with a corresponding diagnosis of a solid malignancy. The primary outcome was 30-day unplanned readmission. We tested five supervised Machine Learning algorithms to develop predictive models: a "48-hour" model, which used data from the first 48 hours after admission, and a "discharge" model, which used data from the entire hospitalization. The optimal models were selected based on their median performance (area under the ROC curve [AUC], precision, recall, F1, and Fβ-Score). Model explainability was assessed using SHAP scores (SS).

**Results:**

We analyzed 3,728 admissions from 1,573 cancer patients, with an unplanned 30-day readmission rate of 21.13%. The Random Forest algorithm demonstrated the best-balanced performance for the 48-hour model (AUC = 0.70, IQR= 0.68-0.71), whereas LightGBM performed best for the discharge model (AUC = 0.70, IQR= 0.68-0.72). In both models, the top predictor was prior emergency department visits in the past six months (SS=0.074 and 0.053, respectively). The top 10 predictors included: laboratory values (including lymphocyte count, Gamma-glutamyl transferase, Red Cell Distribution Width, hemoglobin, sodium, monocyte count, hematocrit, and urea), vital signs, and emergency index admission in both models, with colon cancer ranking among the top predictors in the 48-hour model.

**Conclusion:**

The models underscored the significance of prior emergency department visits, laboratory values, and vital signs as key predictors of 30-day unplanned readmission. The timing of laboratory and vitals influenced their predictive strength. Implementing these models could help providers develop personalized care strategies to reduce readmission risk. External validation is needed.

## Highlights

30-day unplanned readmission rate was 21.13% across the cohort.

Random Forest and LightGBM showed the best-balanced model performance.

Prior Emergency Department visits, labs, and vital signs were key readmission predictors.

Both models achieved median AUC 0.70 with consistent performance.

## In Brief

Stabellini et al. developed Machine Learning models (48-hour and discharge) to predict 30-day unplanned readmissions in cancer patients at a private Brazilian hospital. Analyzing 3,728 admissions, Random Forest and LightGBM performed best (AUC 0.70). Prior Emergency Department visits, vitals, and labs were key predictors, offering a promising tool for targeted patient follow-up.

## Graphical abstract

**Figure:**
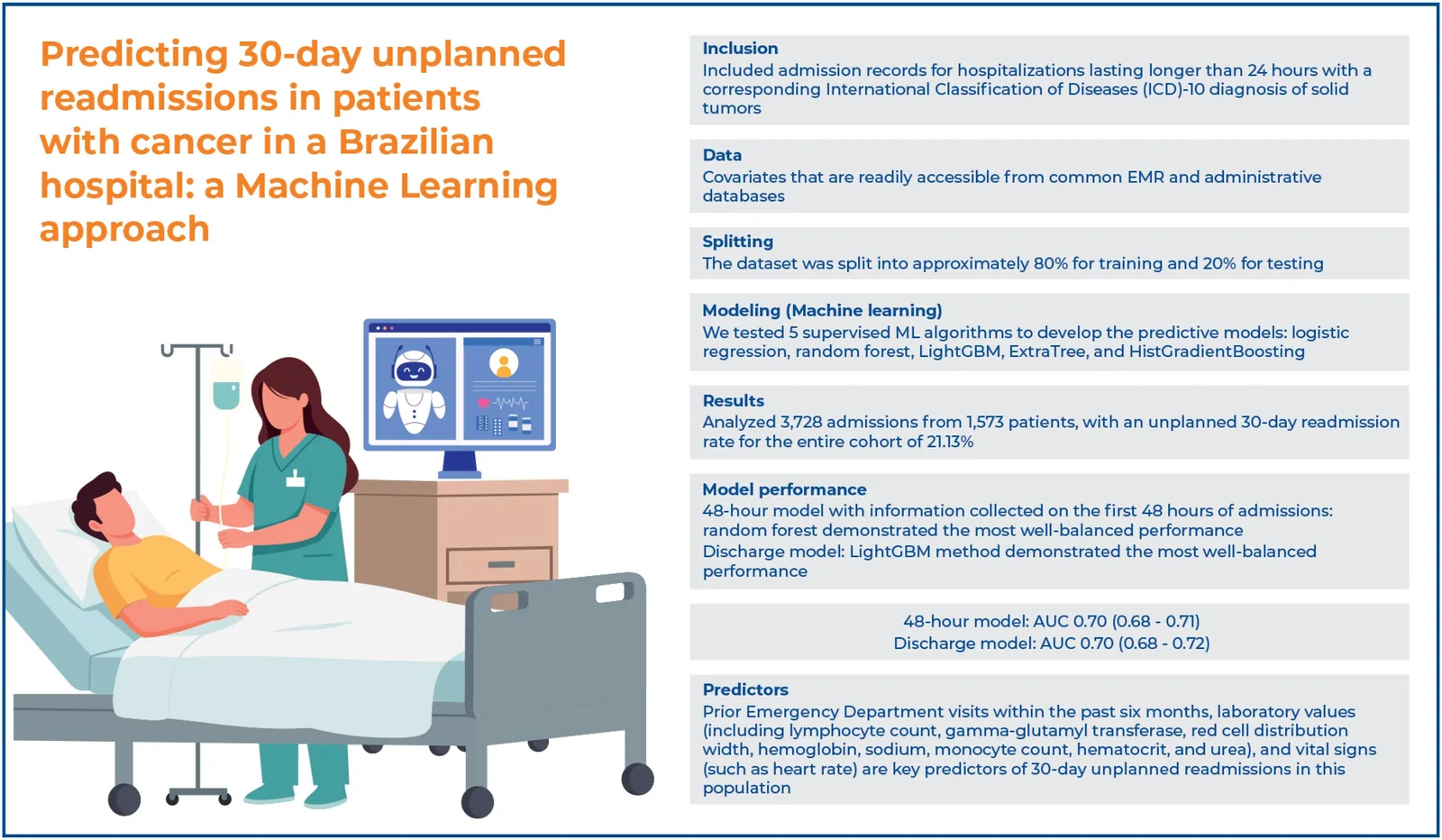

## INTRODUCTION

Cancer ranks as either the first or second leading cause of death in individuals under 70 years of age in 112 out of 183 countries, contributing significantly to the global burden of Disability-Adjusted Life Years, as reported by the World Health Organization.^(1-3)^ In 2020, approximately 19.3 million new cancer cases and nearly 10.0 million cancer-related deaths were recorded worldwide.^(1)^ In Brazil, the largest country in South America and the fifth largest nation globally, with an estimated population of 203.1 million, there were approximately 704,000 new cancer cases in 2023.^(4,5)^

The growing demand for cancer services, the increasing complexity of treatments, and an aging population are driving the rising costs of cancer care.^(6,7)^ Inpatient hospitalizations such as unplanned readmissions represents a major component of the cost of cancer care, as patients with cancer exhibit higher rates of hospitalization and unplanned readmissions compared to the general population.^(8-12)^ In the United States (US) healthcare system, unplanned readmissions in the general population alone are estimated to incur expenses ranging from 15 to 20 billion dollars annually.^(13)^ Cancer itself and the complications arising from anti-cancer treatments can pose various post-discharge challenges.^(14)^ Hospitalized oncology patients, often older, exhibit locally advanced or metastatic disease, malnutrition, substantial symptom burden, varying degrees of dependency, and multiple comorbidities.^(14-16)^ Consequently, reducing unplanned hospital readmissions has the potential to alleviate healthcare costs, enhance healthcare quality, and improve the overall patient experience.^(17,18)^

Prediction represents an effective approach to mitigate unplanned readmissions, and while validated methods such as the LACE and HOSPITAL scores exist for the general population, they demonstrate limited performance when applied to patients with cancer.^(19-22)^ Consequently, there is a need for cancer-specific stratification tools to assess readmission risk. To address this, recent advancements in the field have embraced the utilization of Machine Learning (ML) techniques, which possess the capability to handle the growing volume of diverse healthcare data and seamlessly integrate with electronic medical records.^(23-28)^ Machine learning models have demonstrated superior performance compared to conventional models commonly employed for the general population.^(23-28)^

Our group has recently developed a cancer-specific ML model, designed and validated using data from a large hybrid academic-community practice in the US, that has been shown to perform well in predicting unplanned 30-day readmissions.^(29)^ However, to the best of our knowledge, there is currently a lack of models specifically developed or validated for middle-income countries. We hypothesize that many of the factors identified in our US-based model are also significant predictors in the Brazilian population.

## OBJECTIVE

Therefore, the main objective of this study is to construct machine learning models (a "48-hour" model, which includes data from the first 48 hours after admission, and a "discharge" model, which includes data from the entire hospitalization) to predict 30-day unplanned readmissions in patients with cancer treated at a private hospital in Brazil.

## METHODS

### Study setting

The study was conducted at *Hospital Israelita Albert Einstein*, located in São Paulo, Brazil. Einstein is a 600-bed facility that provides comprehensive care, ranging from secondary to quaternary services, within the private healthcare sector.

### Data source and study design

This retrospective cohort study utilized data extracted from electronic medical records (EMR), information sourced from the oncology department, and administrative database records pertaining to medical admissions at Einstein spanning from July 2018 to August 2023.

We included admission records for hospitalizations lasting longer than 24 hours with a corresponding International Classification of Diseases (ICD)-10 diagnosis of solid tumors. Admissions of patients under the age of 18 years, transfers to other facilities, voluntary discharges, in-hospital deaths, and missing 30-day readmission data were excluded (Table 1S, Supplementary Material). Each hospitalization was treated as an independent index admission. Thus, a single patient with multiple admissions may contribute multiple rows to the dataset, with each row corresponding to a distinct hospitalization.

The Institutional Ethics Committee approved the study protocol of the *Hospital Israelita Albert Einstein*, CAAE: 54221421.1.0000.0071; #5.685.205, waiving the informed consent requirement due to the low confidentiality breach risk, further reduced by data pseudonymization.

### Outcome

The primary outcome was a 30-day unplanned hospital readmission, defined as a nonelective readmission (therefore, a hospitalization categorized as trauma, emergency, or of an urgent nature in the EMR) that occurred within 30 days from the last discharge.^(29)^

### Covariates

With an aim to develop generalizable models, we incorporated an array of covariates that are readily accessible from common EMR and administrative databases. These covariates encompassed a diverse spectrum, including sociodemographic parameters, admission-specific details, vital sign recordings, prognostic risk scores, diagnostic tests, and medical procedures conducted, blood test results, and information spanning the six months antecedent to the index admission. Admission-specific covariates included the cause of admission, admission categorization, ICD-10 diagnostic codes, vital sign profiles, and other pertinent factors (Table 2S, Supplementary Material).

Continuous and integer covariates were summarized using statistical metrics including mean, standard deviation, median, and minimum and maximum values. For categorical variables, we used one-hot encoding to convert them into dummy variables.^(30)^

### Statistical analysis

The data were stratified based on the occurrence of 30-day unplanned readmissions and were presented as absolute values and percentages for categorical variables, together with mean and standard deviation (SD) for normally distributed continuous variables, and median and interquartile range (IQR) for non-normally distributed continuous variables. Categorical variable comparisons were conducted using the Pearson χ^2^ test. Data distribution assumptions for continuous variables were checked through histograms and the Kolmogorov-Smirnov test. Subsequently, Student's *t*-tests were applied for normally distributed factors, and nonparametric Kruskal-Wallis tests were used for non-normally distributed factors.

Covariates that exhibited constant values (with >95% of passages having identical values), continuous variables with more than 20% missing data, categorical variables with more than 2% missing values, and outliers (identified through histograms and box plots, based on clinical implausibility) were excluded from the analysis. A p<0.05 was considered statistically significant.

### Data split

Data stratification was performed based on the study outcome, using patient records for this purpose. To maintain data integrity and prevent any risk of data leakage, we ensured that a patient's record did not concurrently appear in both the training and testing datasets.^(31)^ In other words, if a patient had multiple admissions, each distinct encounter was exclusively assigned to either the training or testing dataset, but not both. Subsequently, the data was randomly divided to ensure that the distribution across two or more datasets accurately represented the overall dataset.

The dataset was split into approximately 80% for training and 20% for testing. The encounters and their corresponding patient records used for testing were consistent for both the 48-hour and discharge models. During the modeling phase, we implemented a repeated stratified cross-validation strategy to fine-tune the readmission models.^(32,33)^ This approach involved dividing the training dataset into "k" parts or "folds" and repeating the process "n" times. In each iteration, the model is trained on k-1 parts and validated on the remaining fold. In total, we employed five folds and ten repetitions, resulting in 50 iterations.

### Algorithms, and hyperparameter optimization

We tested five supervised ML algorithms to develop the predictive models: logistic regression, random forest (an ensemble of decision trees known for its robustness and ability to prevent overfitting), LightGBM (an optimized Gradient Boosting implementation designed for memory efficiency and handling large datasets), ExtraTree (similar to random forest but with an extra level of randomness in node split point selection), and HistGradientBoosting (an efficient variant of Gradient Boosting using histograms to discretize the input space, particularly suitable for large datasets).^(34)^

Each algorithm has specific hyperparameters (predefined settings guiding the algorithm's training process) that need optimization to enhance model accuracy and efficiency.^(35)^ Manually selecting the ideal hyperparameters can be a complex and time-consuming task due to the wide range of possible combinations. To automate and optimize this process, we used the NGOpt optimization algorithm, which conducts a structured search in the hyperparameter space to find the combination maximizing the model's performance.^(36)^ The algorithm employs an iterative approach that integrates Bayesian estimation, gradient, and evolutionary strategies, allowing it to dynamically adapt and effectively explore and adjust hyperparameters.^(36)^

Our optimization criterion was the Fβ-Score, with β=2.^(37)^ We chose this metric due to the data imbalance in the outcome. The value of β=2 was selected to give more weight to sensitivity (recall) at the expense of precision. This decision prioritizes the correct identification of all readmission cases (increasing recall), even if it may result in more false positives (decreasing precision). This approach is particularly important in medical contexts, where it is preferable to err on the side of caution, generating false positives, rather than missing cases, which would result in false negatives.^(29,38)^

### Feature selection

The goal of this stage was to identify and retain variables with the highest predictive relevance, eliminating those whose contribution to the model is minimal.^(39,40)^ This process also helps reduce the model's complexity, improving interpretability and facilitating the deployment and operationalization process.^(39,40)^ The methodology was applied to each of the study's algorithms.

We used an iterative methodology based on SHAP Values (SHapley Additive exPlanations), which provide a metric of each variable's contribution to the model's predictions.^(41)^ This strategy offers an efficient explanatory insight into the impact of variables on ML model predictions.

The feature selection process was conducted using SHAP values in conjunction with stratified five-fold training. During each modeling iteration, the model was fitted using previously optimized hyperparameters, and the importance of each variable was assessed by averaging the absolute SHAP values on the validation data. Subsequently, the average of these values was calculated for each variable across all iterations. Variables with SHAP values above zero were selected and ranked in descending order of importance. We then implemented an iterative process to assess and remove variables that exhibited high correlation with other more important variables, following the SHAP value ranking. The variable correlation was determined using the Pearson coefficient for numerical variables and the Cramér-V coefficient for categorical variables. The correlation threshold was 0.8. Variables that displayed a correlation above this threshold with more important variables according to SHAP values were removed from the model.

Next, we applied the Recursive Feature Elimination method, using the average of absolute SHAP values to evaluate variable importance.^(42,43)^ In this iterative method, the model was initially fitted with all variables, and after repeated five-fold execution, the removal of the variable with the lowest average SHAP value was considered. This removal was only executed if it resulted in an improvement in the average Fβ-Score metric. The process continued until the removal of additional variables no longer resulted in an improvement in the Fβ-Score.

After concluding the variable selection process, we conducted a second hyperparameter optimization, now with the reduced set of variables. This final step aimed to enhance the calibration of hyperparameters for the set after feature selection, maximizing the model's performance.

### Performance metrics and explainability

The performance of the models was analyzed by applying multiple evaluation metrics, all calculated individually for each iteration during the cross-validation process, as well as for the isolated test set at the beginning of the study.^(44,45)^

Firstly, we used Precision, which measures the proportion of positive predictions correctly identified by the model.^(44)^ We followed this with the assessment of Recall, which expresses the proportion of true positive cases correctly identified by the model.^(44)^ To consolidate these two metrics, we calculated the F1-Score, which is the harmonic mean of Precision and Recall.^(44)^ The F1-Score provides a balanced performance measure in situations where classes are unevenly distributed. Recognizing the importance of placing more emphasis on Recall in our context, we also computed the Fβ-Score, with β=2 (implying that Recall is twice as important as Precision in the harmonic mean).

To assess the trade-off between Precision and Recall, we turned to the PRAUC metric (Area under the Precision-Recall Curve).^(38)^ A larger area under this curve indicates a better balance between Precision and Recall. Additionally, we used the ROC-AUC (Area under the Receiver Operating Characteristic Curve) to understand the model's ability to distinguish between readmission and non-readmission.^(45,46)^ A higher value for this metric indicates greater effectiveness of the model in class differentiation.^(46)^

Finally, we calculated the Brier Score to quantify the accuracy of the probabilities predicted by the model.^(45)^ This score measures the discrepancy between predicted probabilities and actual results, with a lower score indicative of better predictions.^(45)^

For metrics that depend on a threshold (Precision, Recall, F1, and Fβ-Score) to be calculated, the cutoff was determined using the Youden method.^(47)^ The Youden method is used to determine the cutoff that optimizes the balance between Sensitivity (Recall) and Specificity in the ROC curve.^(47)^

The optimal models for both the "48 hours" and "discharge" predictions were selected by balancing the best median performance across all evaluated metrics for each ML method. Model explainability was assessed using SHAP scores (SS), which quantify the contribution of each feature to an individual patient's predicted risk.^(41)^ In simple terms, each prediction starts from a baseline value, and the SHAP scores indicate how much each feature increases or decreases the predicted risk relative to that baseline.^(48)^ Mathematically, a prediction, *f*(*x*), can be expressed as a fixed base value plus the sum of the SHAP values: f(x)=base value+ΣSHAP values ^(48)^ For example, if the baseline predicted risk of readmission is 10%, a patient with a history of multiple emergency visits might have a SHAP score of +15% for that feature, increasing their predicted risk to 25%. Conversely, a feature with a SHAP score of −5% would reduce the predicted risk to 20%.

In addition, we performed Decision Curve Analysis to assess the clinical utility of each selected model across a range of threshold probabilities, with a clinical focus on identifying patients at higher risk for the outcome. Given this objective, we prioritized higher sensitivity to minimize missed cases. Furthermore, model performance was evaluated on an independent holdout test set following probability calibration.

### Software and packages

The study was performed in Python 3.10.6 with the following libraries: sickit-learn 1.2.2, scipy 1.10, LightGBM 3.3.5, XGBoost 1.7.5, Nevergrad v0.11.0 and SHAP 0.41.^(49)^

## RESULTS

### Population

We analyzed 3,728 admissions from 1,573 patients (Table 1; Figure 1). The median age was 65 years (IQR, 54-76). Most admissions were from male cancer patients (51%). The median Charlson comorbidity score was 5 (IQR 3-6). The most frequent solid tumor diagnosis was gastrointestinal tract cancer (32.38%). Since their cancer diagnosis, 67.74% of patients received radiotherapy, whereas 84.53% underwent chemotherapy. Most admissions were classified as urgent (51.72%). The median length of stay was four days (IQR 2-8). The unplanned 30-day readmission rate for the entire cohort was 21.13%.

**Table 1:** Patient characteristics stratified by the outcome of 30-day unplanned readmission

Characteristic		Without 30-day unplanned readmission	With 30-day unplanned readmission	p value
		n=2,940 (78.9%)	n=788 (21.1%)	
Age (in years), Mean±SD		64.05 ± 15.36	65.09 ± 14.81	0.08
Male, n (%)		1,501 (51.1)	405 (51.4)	0.89
Admission reason, n (%)				
	Clinical	2,154 (73.3)	629 (79.8)	<0.001
	Surgical	786 (26.7)	159 (20.2)	
Urgent admission, n (%)		1,369 (46.6)	559 (70.9)	<0.001
Length of stay (in days), Median [IQR]		4.00 [2.00-7.00]	5.00 [3.00-10.00]	<0.001
Length of stay of previous admissions (>24h) (in days), Median [IQR]		0.00 [0.00-4.00]	3.00 [0.00-6.00]	<0.001
Number of previous hospitalizations (>24h), Median [IQR]		0.00 [0.00-2.00]	1.00 [0.00-2.00]	<0.001
Number of ED visits in the last 6 months, Median [IQR]		0.00 [0.00-1.00]	1.00 [0.00-2.00]	<0.001
Charlson comorbidity index, Median [IQR]		5.00 [3.00-6.00]	5.00 [3.75-7.00]	<0.01
LACE index, Median [IQR]		9.00 [7.00-12.00]	12.00 [9.00-14.00]	<0.001
MEWS Score, Median [IQR]		1.00 [1.00-1.00]	1.00 [1.00-1.00]	<0.001
Head/Neck cancer, n (%)		115 (3.9)	25 (3.2)	0.388
Digestive organs neoplasms, n (%)		917 (31.2)	290 (36.8)	0.003
Respiratory System Neoplasms, n (%)		351 (11.9)	106 (13.5)	0.276
Bone and Joint Cancer, n (%)		67 (2.3)	4 (0.5)	0.002
Skin cancer, n (%)		72 (2.4)	24 (3.0)	0.417
Soft tissue and mesothelial tissue neoplasms, n (%)		100 (3.4)	25 (3.2)	0.837
Breast and female genital organs cancer, n (%)		622 (21.2)	137 (17.4)	0.022
Male genital organs cancer, n (%)		193 (6.6)	43 (5.5)	0.293
Urinary tract neoplasms, n (%)		178 (6.1)	41 (5.2)	0.41
Eye, brain, and CNS neoplasms, n (%)		153 (5.2)	50 (6.3)	0.24
Endocrine glands neoplasms, n (%)		54 (1.8)	10 (1.3)	0.35
Secondary respiratory and digestive organ cancer, other sites and unspecified (C78-C80), n (%)		85 (2.9)	16 (2.0)	0.23
Chemotherapy, n (%)		2,532 (86.1)	693 (87.9)	0.20
Radiotherapy, n (%)		2,347 (79.8)	670 (85.0)	0.001

The p-values correspond to statistical comparisons between the group with the outcome and the group without.

ED: emergency department; SD: standard deviation; IQR: interquartile range; CNS: central nervous system.

**Figure 1:**
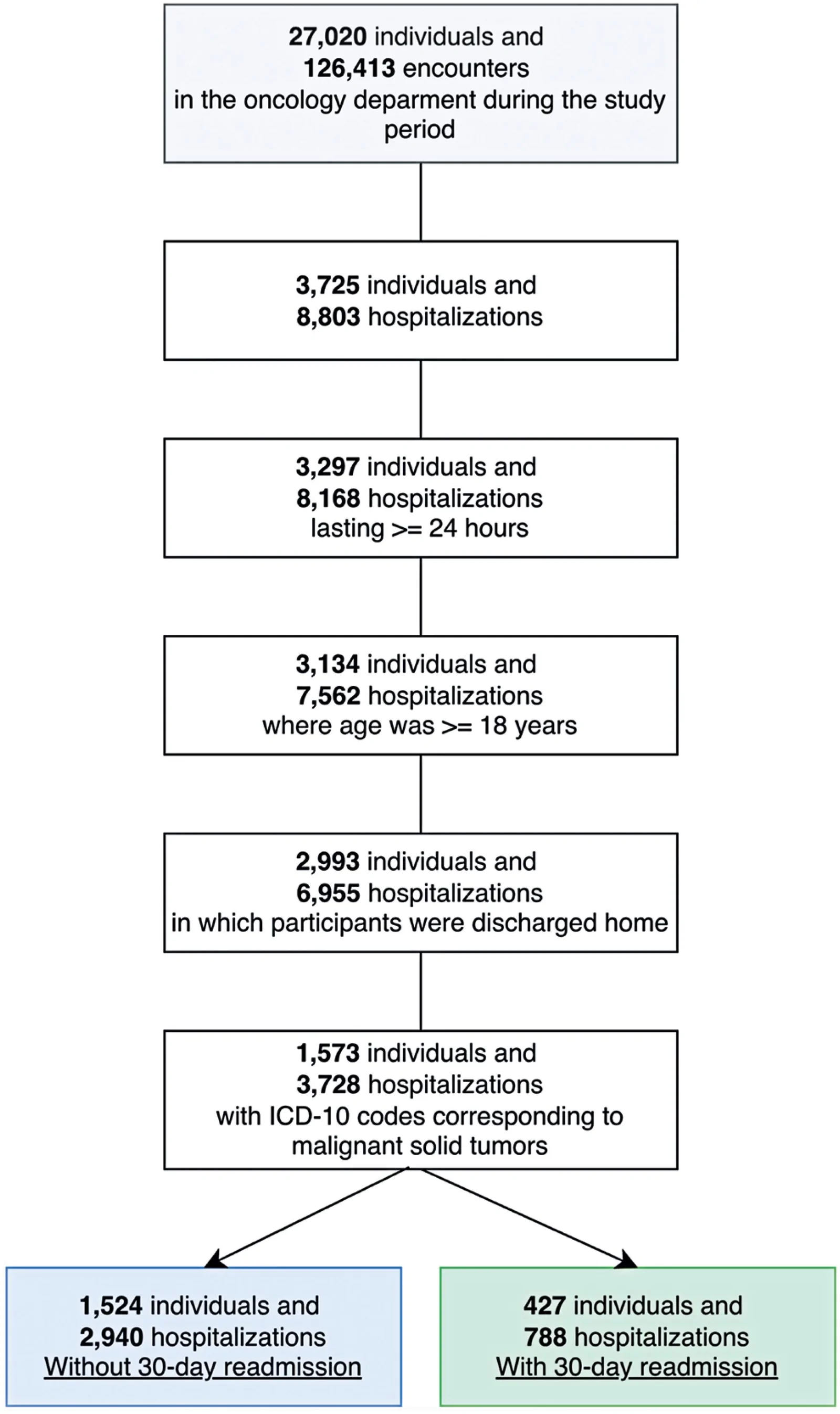
CONSORT diagram illustrating patient exclusions based on each criterion, leading to the final analyzed cohort

When comparing patient characteristics (Table 1), we found that, considering data obtained up to the index admission, patients who experienced a 30-day unplanned readmission, compared with those who did not, exhibited the following: lower rates of surgical admissions (20.2% *versus* 26.7%, p<0.001), higher rates of urgency admissions (70.9% *versus* 26.7%, p<0.001), a longer length of stay in days (median of 5 [IQR 3-10] *versus* 4 [IQR 2-7], p<0.001), an extended length of stay in previous admissions (median of 3 [IQR 0-6] *versus* 0 [IQR 0-4], p<0.001), a higher median of previous admissions (1 [IQR 0-2] *versus* 0 [IQR 0-2], p<0.001), a higher median of visits to the emergency department (ED) in the last six months (1 [IQR 0-2] *versus* 0 [IQR 0-1], p<0.001), higher median Charlson scores (5 [IQR 3.75-7] *versus* 5 [IQR 3-6], p=0.01), higher median LACE index (12 [IQR 9-14] *versus* 9 [IQR 7-12], p<0.001), higher rates of digestive organ neoplasia (36.8% *versus* 31.2%, p=0.003), and higher rates of radiotherapy (85% *versus* 79.8%, p=0.001).

### Model with covariates from the first 48 hours after admission (48-hour model)

The Random Forest method demonstrated the most well-balanced performance for the 48-hour model (Table 2 and Table 3S, Figure 1S, Figure 2S, Supplementary Material).

**Table 2:** Performance metrics of the ML algorithms that performed best in predicting 30-day unplanned readmission in the "48-hour" model and the "discharge" model

	48-hour model	Discharge model
Top performing ML approach	Random Forest	LightGBM
AUC - median (IQR)	0.70 (0.68-0.71)	0.70 (0.68-0.72)
PRAUC - median (IQR)	0.38 (0.35-0.51)	0.37 (0.34-0.40)
Precision - median (IQR)	0.33 (0.30-0.36)	0.33 (0.31-0.35)
F1 score - median (IQR)	0.44 (0.42-0.46)	0.45 (0.43-0.47)
Fβ score - median (IQR)	0.56 (0.54-0.58)	0.59 (0.56-0.60)
True positive rate - median (IQR)	0.70 (0.62-0.77)	0.74 (0.67-0.76)
True negative rate - median (IQR)	0.64 (0.55-0.71)	0.61 (0.55-0.68)
False negative rate - median (IQR)	0.30 (0.23-0.38)	0.26 (0.24-0.33)
False positive rate - median (IQR)	0.36 (0.29-0.45)	0.39 (0.32-0.45)

AUC: area under the curve; PRAUC: area under the precision-recall curve; IQR: interquartile range.

The top 10 predictors of an unplanned 30-day readmission, ranked by the Random Forest 48-hour model (Figure 2), included the following: (a) ED visits in the last six months (SS=0.074); (b) Emergency or urgency index admission (SS=0.049); (c) Maximum lymphocyte value (SS=0.043); (d) Mean sodium value (SS=0.023); (e) Mean Red Cell Distribution Width (RDW) value (SS=0.019); (f) Mean lymphocytes value (SS=0.012); (g) Mean urea value (SS=0.011); (h) Minimum hemoglobin value (SS=0.008); (i) Minimum heart rate value (SS=0.006); (j) Colorectal cancer diagnosis (SS=0.006).

**Figure 2:**
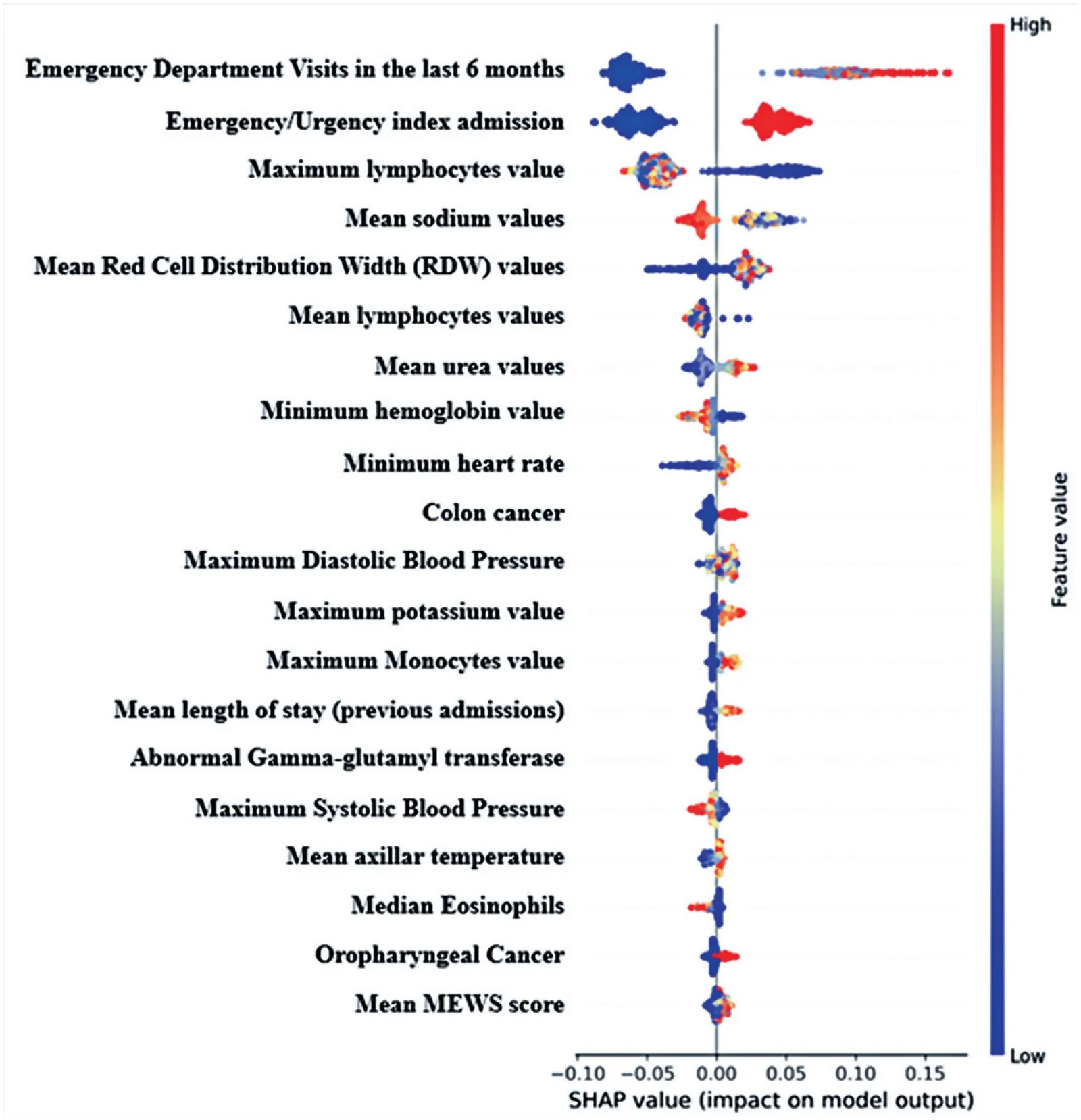
SHAP (SHapley Additive exPlanations) analysis from the "48-hour" Random Forest model, ranking the top-20 predictors of 30-day Unplanned Readmission in patients with cancer. Positive SHAP values (on the right side of the line) favor the outcome. The color gradient indicates the feature values/categories

### Model with covariates from the entire admission (discharge model)

The LightGBM method demonstrated the most well-balanced performance for the discharge model (Table 2 and Table 3S, Figures 1S and 2S, Supplementary Material).

The top 10 predictors of an unplanned 30-day readmission, ranked by the LightGBM discharge model, were (Figure 3): (a) ED visits in the last six months prior to index admission (SS=0.053); (b) Mean lymphocyte values (SS=0.043); (c) Absolute Lymphocytes value at discharge (SS=0.031); (d) Emergency or urgency index admission (SS=0.029); (e) Mean Gamma-glutamyl transferase (GGT) values (SS=0.028); (f) Maximum RDW value (SS=0.001); (g) Absolute Monocyte value at discharge (SS=0.001); (h) GGT outside the normal range (SS=0.001); (i) Minimum hematocrit value (SS=0.001); (j) Total number of GGT values outside the normal range (SS=0.001).

**Figure 3:**
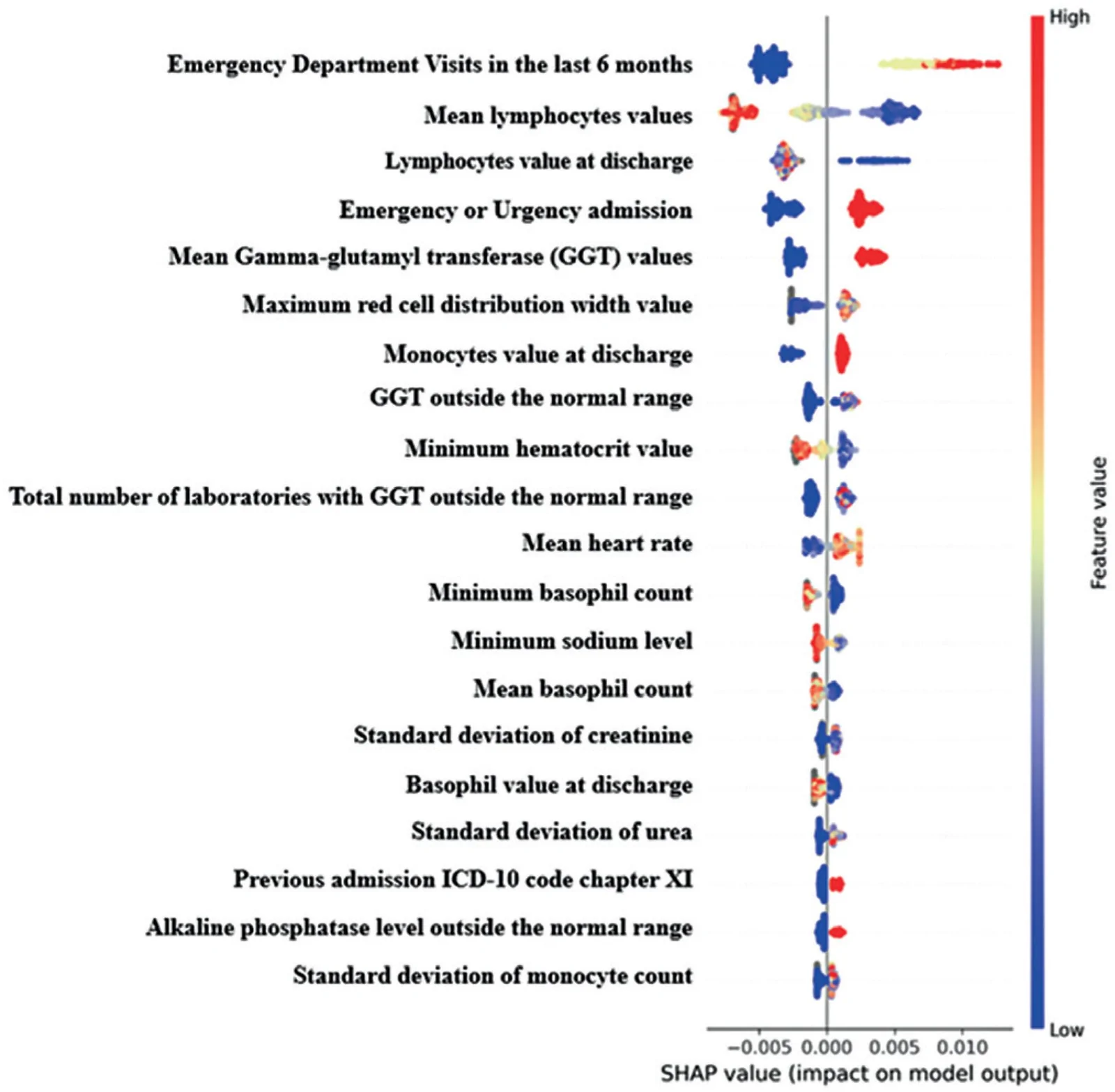
SHAP (SHapley Additive exPlanations) analysis from the "discharge" LightGBM model, ranking the top-20 predictors of 30-day Unplanned Readmission in patients with cancer. Positive SHAP values (on the right side of the line) favor the outcome. The color gradient indicates the feature values/categories

## DISCUSSION

To the best of our knowledge, this is the first study to build two distinct ML models for predicting 30-day unplanned readmissions in patients with cancer in Brazil or in other middle-income countries. Using data from a private institution in the country, we developed a "48-hour" model and a "discharge" model. Our results show that prior ED visits within the past six months, laboratory values (including lymphocyte count, GGT, RDW, hemoglobin, sodium, monocyte count, hematocrit, and urea), and vital signs (such as heart rate) are key predictors of 30-day unplanned readmissions in this population. These two predictive models demonstrated good performance and could be utilized to help identify cancer patients at higher risk of unplanned readmissions, who might benefit from targeted monitoring and closer follow-up.

This project builds upon a 30-day unplanned readmission model developed and published by our group for the US population, while adapting it to the Brazilian context.^(29)^ Brazil and the US have distinct cancer epidemiology and different profiles in terms of social determinants of health (SDOH), resulting in Brazilian cancer patients facing distinct barriers and adversities compared to their US counterparts.^(50-52)^ Unlike the US model, this current Brazilian model did not include SDOH data due to its unavailability, though the US model showed that including SDOH data did not improve prediction accuracy.^(29)^ While the US model incorporated data from the entire index admission for each patient, the Brazilian models focused on admissions rather than individuals and were stratified into two models: one using data from the first 48 hours and the other using data from the admission time only.^(29)^ The ML approach that performed best in the Brazilian discharge model was LightGBM, which also had the top performance in the US model.^(29)^ Both models identified prior unplanned readmissions and specific lab data (anion gap, hematocrit, platelets, hemoglobin, sodium, and mean corpuscular hemoglobin) as key predictors of 30-day unplanned readmissions.^(29)^ However, the US model also identified additional predictors, such as the Charlson Comorbidity Index, discharge site, percentage of appointments attended, and the number of medications administered during admission.^(29)^ Furthermore, the US model demonstrated better performance with an AUC of 0.80 compared to 0.70 for both the Brazilian discharge and 48-hour models.^(29)^ Nevertheless, these results should be interpreted with caution, as the models were developed using different methodologies and distinct datasets.

In the Brazilian population, older patients with cancer have been shown to have an approximately three-fold higher risk of 30-day unplanned readmission, and cancer diagnosis was the only significant predictor of this outcome in multivariable regression models.^(53)^ The same study reported a 30-day unplanned readmission rate of 12.5% in the analyzed population, which included both cancer and non-cancer patients.^(53)^ Among patients with cancer, a 30-day readmission rate of 33.5% was reported at a Brazilian tertiary cancer center.^(54)^ Our study reports a 30-day unplanned readmission rate of 21.1%, consistent with the 21.3% reported in our US model study, and aligns with the literature, which indicates that unplanned readmission rates in cancer patients can reach as high as 30%.^(29)^

Unplanned readmissions in those diagnosed with cancer are influenced by demographic and clinical factors, including age, race, ethnicity, insurance status, comorbidities, cancer type, cancer stage, mental health conditions, and length of stay.^(55-57)^ A retrospective cohort study conducted at a tertiary cancer center in São Paulo, Brazil, found that among patients diagnosed with solid tumors, unplanned admissions were characterized by a mean age of 62 years, with the majority being female (51.6%).^(54)^ These patients had a median Charlson Comorbidity Index of 6 (IQR 4-6), and unplanned admissions were more common among those with breast cancer, followed by colon cancer, and among patients undergoing systemic treatment.^(54)^ The study also revealed that the most common symptoms at admission were pain, nausea, and dyspnea, with a median length of stay of six days (IQR 3-10), and approximately 50% of patients were readmitted within 90 days.^(54)^ Our demographic findings are consistent with these results, showing similar patterns in patients with a 30-day unplanned readmissions, particularly in terms of age, Charlson score, length of stay, and cancer type.

Despite evidence to the contrary, neither of our models suggested that demographic factors influence unplanned hospital readmissions because demographic information was not among the key predictors.^(55-57)^ Instead, ED visits in the last six months, laboratory values, and vital signs emerged as the top predictors of 30-day unplanned readmissions in cancer patients in Brazil. The lead predictive performance of ED visits in the last six months may reflect "high-utilizers" or "super-utilizers" patients with high needs and extensive healthcare utilization, accounting for a significant portion of ED visits, hospitalizations, and readmissions.^(58)^ This may also represent patients whose cancer is progressing, worsening their health and increasing their susceptibility to conditions that lead to admissions and subsequent unplanned readmissions.^(59)^ The importance of laboratory values and vital signs aligns with literature showing that, in patients receiving systemic cancer treatment, abnormal lab results are linked to a higher risk of hospitalization.^(16,60,61)^ Our study adds to this by demonstrating that the timing of when a lab result or vital sign is recorded during admission can influence its predictive weight for a 30-day unplanned readmission. It is interesting that RDW was identified by the model as a significant laboratory factor given that prior research has shown RDW values to have a negative correlation with lifespan in murine studies evaluating the impact of dietary restrictions on lifespan.^(62)^

It is noteworthy that Brazil has a dual healthcare system composed of the universal public system, the *Sistema Único de Saúde* (SUS), and a supplementary private sector.^(63)^ Approximately 70-75% of the Brazilian population relies exclusively on SUS for healthcare services, whereas only about 25% have access to private health insurance, which is generally associated with people holding higher socioeconomic status.^(64-66)^ Consequently, patients treated in private hospitals such as Einstein may differ from the broader Brazilian population in socioeconomic characteristics and access to healthcare resources. Compared with many public or community hospitals across Brazil that predominantly serve SUS-dependent populations, Einstein functions as a high-complexity center with greater technological resources and access to specialized care. In this regard, the institutional structure and clinical capabilities of Einstein may resemble those of large academic medical centers in high-income countries, including the US.^(67)^ Therefore, the results of this study should be interpreted with caution in the context of other institutions or populations.

Our study has several limitations. Despite employing multiple approaches to minimize model bias (such as data splitting, cross-validation, testing multiple ML methodologies, Recursive Feature Elimination) the models were not externally validated. Additionally, although we used the Fβ-Score, the imbalance between readmissions and non-readmissions may have biased record selection (reflecting a high rate of "high-utilizers") and inflated model performance. Social and bureaucratic factors (e.g., marital status, social support, health insurance, and reimbursement policies) may influence decisions to admit or readmit patients and were not accounted for in our models. The single-center, private-practice setting may also have biased the results toward a population with better socioeconomic and health conditions. Furthermore, some patients may have been lost to follow-up or sought care at other institutions. The use of retrospective data limited the available covariates, and some important factors, such as SDOH, were either unavailable or had a high rate of missing data. The use of EMR data may contain inconsistencies and missing information related to the data entry process and procedures (e.g., regarding staging information, which could not be included in the study due to the high rate of missingness [>90%]). The use of ICD codes to define cancer types may lead to underrepresentation and inconsistencies.

## CONCLUSION

This study developed two distinct ML models (the "48-hour model" [which incorporates data from the first 48 hours of admission], and the "discharge model" [which includes data from the entire admission]) to predict 30-day unplanned readmissions in cancer patients in Brazil, using commonly available data. The models showed good performance and highlighted the importance of previous emergency department visits, laboratory values, and vital signs as predictors of this outcome. Additionally, the timing of laboratory or vital signs impacted their predictive weight. From a clinical practice perspective, while external validation is needed, these models could be implemented (as standalone tools or integrated into electronic medical records) to identify patients at high risk for 30-day unplanned readmission. This would enable providers to develop personalized care strategies to mitigate risk, such as closer follow-up through telemedicine, the use of remote monitoring tools, and/or multidisciplinary home care. Future research should focus on the external validation of our models, prospective design and implementation, multi-center studies, larger cohorts, the inclusion of a broader range of covariates, the integration of social determinants of health data, and the evaluation of implementation strategies.

## Data Availability

The *Hospital Israelita Albert Einstein* database is available at the institution and has access restricted to researchers with IRB approval.
