# Supplementary material for: Predicting 30-day unplanned readmissions in patients with cancer in a Brazilian hospital: a Machine Learning approach
Source: Einstein (Sao Paulo). 2026 Sep 2;24:eAO2210. doi: 10.31744/einstein_journal/2026AO2210 (PMC13682789; doi:10.31744/einstein_journal/2026AO2210)
Supplement: Supplementary file 1 [file 2317-6385-eins-24-eAO2210-Suppl01.pdf]

SUPPLEMENTARY MATERIAL

Predicting 30-day unplanned readmissions in patients with cancer in a Brazilian hospital: a Machine Learning approach

Nickolas Stabellini, Marilia Melo Favalesso, Claudia Szlejf, Cesar Gomes Miguel, Roberto Carmagnani Pestana, Hanna Kim, Gabriela de Carvalho Ferreira, Kristin A. Waite, Alberto J. Montero, Jill S. Barnholtz-Sloan, Edson Amaro Junior, Nelson Hamerschlak

DOI: 10.31744/einstein_journal/2026AO2210

Table 1S. International Classification of Diseases (ICD) Chapter 10 codes used to define the various cancer types (solid tumors) included in this study

Cancer Type	ICD-10 corresponding codes*
Head/Neck Cancer	C00.XX-C14.XX
Digestive Organs Neoplasms	C15.XX-C26.XX
Liver and Intrahepatic Bile Duct Neoplasms	C22.XX
Respiratory System Neoplasms	C30.XX-C39.XX
Bone and Joint Neoplasms	C40.XX-C41.XX
Skin Neoplasms	C43.XX-C44.XX
Mesothelial and Soft Tissue Neoplasms	C45.XX-C49.XX
Breast and Female Genital Organs Neoplasms	C50.XX-C58.XX
Male Genital Organs Neoplasms	C60.XX-C63.XX
Urinary Tract Neoplasms	C64.XX-C68.XX
Eye, Brain, and CNS Neoplasms	C69.XX-C72.XX
Endocrine Gland Neoplasms	C73.XX-C76.XX
Unspecified Neoplasm	C80.XX
Secondary Cancers of Respiratory and Digestive Organs, Other Sites, and Unspecified	C78.XX-C80.XX

* X represents any integer.

Table 2S. List of covariates included in the study, including their names as shown in the dataset and a description detailing what each covariate refers to

Covariate name in the dataset	Description
25_oh_vitamina_d_ng_ml_21786460_alterado_relativo	25-hydroxyvitamin D (ng/mL) Exams Outside the Reference Range
25_oh_vitamina_d_ng_ml_21786460_total_exms	Total number of 25-hydroxyvitamin D exams (ng/mL)
25_oh_vitamina_d_ng_ml_21786460_total_positivos	Total number of positive results in 25-hydroxyvitamin D exams (ng/mL)
albumina_g_dl_21787332_ult_med_alt	Alteration in the latest measured albumin level (g/dL)
basofilos_µl_21785724_max	Maximum basophil count (µL)
basofilos_µl_21785724_media	Mean basophil count (µL)
basofilos_µl_21785724_mediana	Median basophil count (µL)
basofilos_µl_21785724_min	Minimum basophil count (µL)
basofilos_µl_21785724_ult_med_alt	Alteration in the latest median basophil count (µL)
basofilos_µl_21785724_ult_med_num	Number of measurements for the latest median basophil count (µL)
basofilos_21785664_mediana	Median basophil count (%)
basofilos_21785664_min	Minimum basophil count (%)
basofilos_21785664_ult_med_alt	Alteration in the latest median basophil count (%)
basofilos_21785664_ult_med_num	Number of measurements for the latest median basophil count (%)
basofilos_Âµl_21785724_mediana	Median basophil count (µL)
basofilos_Âµl_21785724_min	Minimum basophil count (µL)

continue...

...Continuation

Table 2S. List of covariates included in the study, including their names as shown in the dataset and a description detailing what each covariate refers to

Covariate name in the dataset	Description
bastonestes_21785160_ult_med_alt	Alteration in the latest median band count
bastonetes_µl_21787276_alterado_relativo	Number of altered results of basophil count (µL) relative to the reference value
bastonetes_µl_21787276_total_exms	Total number of basophil count exams (µL)
bastonetes_µl_21787276_total_positivos	Total number of positive results in basophil count (µL)
be_gasometria_venosa_meq_l_21787274_ult_med_alt	Alteration in the latest measured base excess in venous blood gas analysis (mEq/L)
bilirrubina_direta_mg_dl_21785520_ult_med_alt	Alteration in the latest measured direct bilirubin level (mg/dL)
bilirrubina_indireta_mg_dl_21786074_media	Mean of indirect bilirubin (mg/dL)
bilirrubina_indireta_mg_dl_21786074_mediana	Median of indirect bilirubin (mg/dL)
bilirrubina_indireta_mg_dl_21786074_min	Minimum of indirect bilirubin (mg/dL)
bilirrubina_indireta_mg_dl_21786074_ult_med_alt	Alteration in the latest measured indirect bilirubin level (mg/dL)
bilirrubina_total_mg_dl_21787252_alterado_relativo	Number of altered results of total bilirubin (mg/dL) relative to the reference value
bilirrubina_total_mg_dl_21787252_total_exms	Total number of total bilirubin exams (mg/dL)
bilirrubina_total_mg_dl_21787252_total_positivos	Total number of positive results in total bilirubin (mg/dL)
calc_mews_max	Maximum MEWS (Modified Early Warning Score) calculation value
calc_mews_mediana	Median MEWS score
calc_mews_medio	Mean MEWS (Modified Early Warning Score) calculation value
calc_mews_min	Minimum MEWS score
calc_mews_ult_med	Alteration in the latest measured MEWS (Modified Early Warning Score) calculation value
calcio_ionico_mmol_l_mmol_l_21785564_ult_med_alt	Alteration in the latest measured ionized calcium level (mmol/L)
cancer_secundarios_orgaos_respiratorios_digestivos_outros_locais_nao_especificados	Presence of secondary cancers in respiratory and digestive organs and other unspecified locations
cci_diabetes_sem_comp	Presence of diabetes without complications in Charlson Comorbidity Index
cci_doen_arteri_periferica	Presence of peripheral arterial disease in Charlson Comorbidity Index
cci_doen_hep_cron_leve	Mild liver disease
cci_doen_pulm_cron	Chronic pulmonary disease
cci_insuf_cardiaca	Heart failure
cci_insuf_renal_cci	Moderate to severe chronic kidney disease
charlson_comorb_idx	Charlson Comorbidity Index
chcm_g_dl_21687758_max	Maximum mean corpuscular hemoglobin concentration (CHCM) level (g/dL)
chcm_g_dl_21687758_media	Mean corpuscular hemoglobin concentration (CHCM) level (g/dL)
chcm_g_dl_21687758_mediana	Median mean corpuscular hemoglobin concentration (CHCM) level (g/dL)
chcm_g_dl_21687758_min	Minimum mean corpuscular hemoglobin concentration (CHCM) level (g/dL)
chcm_g_dl_21687758_ult_med_alt	Alteration in the latest mean corpuscular hemoglobin concentration (CHCM) level (g/dL)
chcm_g_dl_21687758_ult_med_num	Number of measurements for the latest mean corpuscular hemoglobin concentration (CHCM) level (g/dL)
cid_atual_c1	Index admission ICD-10 code chapter I
cid_atual_c7	Index admission ICD-10 code chapter VII
cid_atual_c9	Index admission ICD-10 code chapter IX
cid_hist_c1	Previous admission ICD-10 code chapter I
cid_hist_c10	ICD-10 code chapter C10 for previous admission
cid_hist_c11	Previous admission ICD-10 code chapter XI
cid_hist_c13	ICD-10 code chapter C13 for previous admission
cid_hist_c14	Previous admission ICD-10 code chapter XIV
cid_hist_c18	ICD-10 code chapter C18 for previous admission
cid_hist_c19	Previous admission ICD-10 code chapter XIX
cid_hist_c2	Previous admission ICD-10 code chapter II
cid_hist_c21	Previous admission ICD-10 code chapter XXI
cid_hist_c3	ICD-10 code chapter C3 for previous admission
cid_hist_c4	ICD-10 code chapter C4 for previous admission

continue...

...Continuation

Table 2S. List of covariates included in the study, including their names as shown in the dataset and a description detailing what each covariate refers to

Covariate name in the dataset	Description
cid_hist_c5	Previous admission ICD-10 code chapter V
cid_hist_c6	Previous admission ICD-10 code chapter VI
cid_hist_c9	ICD-10 code chapter C9 for previous admission
cirurgia_eletivo_surgical	Indicator for elective surgery during admission
co2_total_gasometria_venosa_mmol_l_21785800_ult_med_alt	Alteration in the latest measured total carbon dioxide in venous blood gas analysis (mmol/L)
creatinina_mg_dl_21686440_mediana	Median creatinine (mg/dL)
creatinina_mg_dl_21686440_min	Minimum creatinine (mg/dL)
creatinina_mg_dl_21686440_ult_med_alt	Alteration in the latest measured creatinine level (mg/dL)
creatinina_mg_dl_21686440_ult_med_num	Number of measurements for the latest measured creatinine level (mg/dL)
creatinina_tlr_poct_mg_dl_25401693_ult_med_alt	Alteration in the latest measured creatinine level by point-of-care testing (mg/dL)
CX_hist	Number of comorbidities
determinacao_de_glicemia_capilar_mg_dl_25287943_mediana	Median capillary blood glucose (mg/dL)
determinacao_de_glicemia_capilar_mg_dl_25287943_min	Minimum capillary blood glucose (mg/dL)
dias_desde_inicio_quimio	Number of days since the start of chemotherapy
dias_desde_inicio_radio	Number of days since the start of radiotherapy
dosagem_de_glicose_mg_dl_21787974_ult_med_alt	Alteration in the latest measured glucose level (mg/dL)
eosinofilos_µl_21787514_max	Maximum eosinophil count (µL)
eosinofilos_µl_21787514_media	Mean eosinophil count (µL)
eosinofilos_µl_21787514_mediana	Median eosinophil count (µL)
eosinofilos_µl_21787514_min	Minimum eosinophil count (µL)
eosinofilos_µl_21787514_ult_med_alt	Alteration in the latest median eosinophil count (µL)
eosinofilos_µl_21787514_ult_med_num	Number of measurements for the latest median eosinophil count (µL)
eosinofilos_21785258_mediana	Median eosinophil count (%)
eosinofilos_21785258_min	Minimum eosinophil count (%)
eosinofilos_21785258_ult_med_alt	Alteration in the latest median eosinophil count (%)
eosinofilos_21785258_ult_med_num	Number of measurements for the latest median eosinophil count (%)
estado_civil	Marital status
fg_msm_cid_inter_ant	Same ICD-10 code chapter from previous admission
fg_msm_fami_cid_inter_ant	Same ICD-10 code chapter from previous admission in family history
fibrinogenio_mg_dl_21785644_alterado_relativo	Number of altered results of fibrinogen (mg/dL) relative to the reference value
fibrinogenio_mg_dl_21785644_total_exms	Total number of fibrinogen exams (mg/dL)
fibrinogenio_mg_dl_21785644_total_positivos	Total number of positive results in fibrinogen exams (mg/dL)
fibrinogenio_none_21785644_alterado_relativo	Number of altered results of fibrinogen (unit unspecified) relative to the reference value
fibrinogenio_none_21785644_total_exms	Total number of fibrinogen exams (unit unspecified)
fibrinogenio_none_21785644_total_positivos	Total number of positive results in fibrinogen exams (unit unspecified)
fio2_gasometria_venosa_none_21785168_alterado_relativo	Number of altered results of venous blood gas oxygenation (unit unspecified) relative to the reference value
fio2_gasometria_venosa_none_21785168_diff_alt	Difference between the current measurement and the last altered measurement of venous blood gas oxygenation (unit unspecified)
fio2_gasometria_venosa_none_21785168_freq_positivos	Frequency of positive results in venous blood gas oxygenation (unit unspecified)
fio2_gasometria_venosa_none_21785168_total_exms	Total number of venous blood gas oxygenation exams (unit unspecified)
fio2_gasometria_venosa_none_21785168_total_positivos	Total number of positive results in venous blood gas oxygenation exams (unit unspecified)
fio2_gasometria_venosa_none_21785168_ult_med_alt	Last altered measurement of venous blood gas oxygenation (unit unspecified)
flag_cirurgia	Surgery performed during admission
flag_emerg_urgencia	Urgency or emergency admission
flag_pandemia_covid	Indicator for COVID-19 pandemic period
flag_tratamento_quimio_ultimos_6m	Underwent chemotherapy in the last 6 months
flag_tratamento_radio_ultimos_6m	Underwent radiotherapy in the last 6 months

continue...

...Continuation

Table 2S. List of covariates included in the study, including their names as shown in the dataset and a description detailing what each covariate refers to

Covariate name in the dataset	Description
flag_uti	Presence of ICU stay
fosfatase_alcalina_u_l_21785844_ult_med_alt	Alteration in the latest measured alkaline phosphatase level (U/L)
freq_card_max	Maximum heart rate
freq_card_mediana	Median heart rate
freq_card_medio	Mean heart rate (bpm)
freq_card_min	Minimum heart rate
freq_card_var	Heart rate variance (bpm)
gama_gt_u_l_21786350_ult_med_alt	Alteration in the latest measured gamma-glutamyltransferase level (U/L)
glicemia_mg_dl_25287943_media	Mean fasting blood glucose (mg/dL)
glicemia_mg_dl_25287943_min	Maximum fasting blood glucose (mg/dL)
glicose_tlr_poct_mg_dl_25401688_alterado_relativo	Number of altered results of glucose point-of-care testing (mg/dL) relative to the reference value
glicose_tlr_poct_mg_dl_25401688_total_exms	Total number of glucose point-of-care testing exams (mg/dL)
glicose_tlr_poct_mg_dl_25401688_total_positivos	Total number of positive results in glucose point-of-care testing (mg/dL)
hcm_g_dl_21687758_media	Mean CMCH (g/dL)
hcm_pg_21687752_mediana	Median corpuscular hemoglobin (HCM) (pg)
hcm_pg_21687752_min	Minimum corpuscular hemoglobin (HCM) (pg)
hcm_pg_21687752_ult_med_alt	Alteration in the latest measured mean corpuscular hemoglobin (HCM) level (pg)
hcm_pg_21687752_ult_med_num	Number of measurements for the latest measured mean corpuscular hemoglobin (HCM) level (pg)
hco3_gasometria_venosa_meq_l_21785036_ult_med_alt	Alteration in the latest measured bicarbonate level in venous blood gas analysis (mEq/L)
hemacias_urina_ml_21785822_ult_med_alt	Alteration in the latest measured red blood cells in urine (mL)
hemacias_x106_ul_21687730_max	Maximum red blood cell count (x10^6/µL)
hemacias_x106_ul_21687730_media	Mean red blood cell count (x10^6/µL)
hemacias_x106_ul_21687730_mediana	Median red blood cell count (x10^6/µL)
hemacias_x106_ul_21687730_min	Minimum red blood cell count (x10^6/µL)
hemacias_x106_ul_21687730_ult_med_alt	Alteration in the latest measured median red blood cell count (x10^6/µL)
hemacias_x106_ul_21687730_ult_med_num	Number of measurements for the latest measured median red blood cell count (x10^6/µL)
hematocrito_21687740_max	Maximum hematocrit level
hematocrito_21687740_media	Mean hematocrit (%)
hematocrito_21687740_mediana	Median hematocrit level
hematocrito_21687740_min	Minimum hematocrit level
hematocrito_21687740_ult_med_alt	Alteration in the latest measured hematocrit level
hematocrito_21687740_ult_med_num	Number of measurements for the latest measured hematocrit level
hemoglobina_g_dl_21687734_max	Maximum hemoglobin level (g/dL)
hemoglobina_g_dl_21687734_media	Mean hemoglobin level (g/dL)
hemoglobina_g_dl_21687734_mediana	Median hemoglobin level (g/dL)
hemoglobina_g_dl_21687734_min	Minimum hemoglobin level (g/dL)
hemoglobina_g_dl_21687734_ult_med_alt	Alteration in the latest measured hemoglobin level (g/dL)
hemoglobina_g_dl_21687734_ult_med_num	Number of measurements for the latest measured hemoglobin level (g/dL)
horas_internacao_lace	Length of stay (hours)
idade	Age
ig_µl_21786312_ult_med_alt	Alteration in the latest measured immunoglobulin level (µL)
ig_21787800_ult_med_alt	Alteration in the latest measured immunoglobulin level
iniciou_quimio	Indicator for the initiation of chemotherapy
iniciou_radio	Indicator for the initiation of radiotherapy
ipf_µl_21786362_ult_med_alt	Alteration in the latest measured immature platelet fraction level (µL)
ipf_21787972_ult_med_alt	Alteration in the latest measured immature platelet fraction level
lactato_venoso_tlr_poct_mg_dl_25401703_ult_med_alt	Alteration in the latest measured venous lactate level by point-of-care testing (mg/dL)

continue...

...Continuation

Table 2S. List of covariates included in the study, including their names as shown in the dataset and a description detailing what each covariate refers to

Covariate name in the dataset	Description
leucocitos_µl_21687720_max	Maximum leukocyte count (µL)
leucocitos_µl_21687720_media	Mean leukocyte count (µL)
leucocitos_µl_21687720_mediana	Median leukocyte count (µL)
leucocitos_µl_21687720_min	Minimum leukocyte count (µL)
leucocitos_µl_21687720_ult_med_alt	Alteration in the latest measured mean leukocyte count (µL)
leucocitos_µl_21687720_ult_med_num	Number of measurements for the latest measured mean leukocyte count (µL)
leucocitos_urina_ml_21785912_ult_med_alt	Alteration in the latest measured leukocytes in urine (mL)
leucocitos_x103_ul_21687712_media	Mean leukocyte count (µL)
leucocitos_x103_ul_21687712_mediana	Median leukocyte count (x10^3/µl)
leucocitos_x103_ul_21687712_min	Minimum leukocyte count (x10^3/µl)
leucocitos_x103_ul_21687712_ult_med_alt	Alteration in the latest measured leukocyte count (x10^3/µL)
leucocitos_x103_ul_21687712_ult_med_num	Number of measurements for the latest measured leukocyte count (x10^3/µL)
linfocitos_µl_21787060_max	Maximum lymphocyte count (µL)
linfocitos_µl_21787060_media	Mean lymphocyte count (µL)
linfocitos_µl_21787060_mediana	Median lymphocyte count (µL)
linfocitos_µl_21787060_min	Minimum lymphocyte count (µL)
linfocitos_µl_21787060_ult_med_alt	Alteration in the latest measured median lymphocyte count (µL)
linfocitos_µl_21787060_ult_med_num	Number of measurements for the latest measured median lymphocyte count (µL)
linfocitos_21786624_media	Mean lymphocyte count (%)
linfocitos_21786624_mediana	Median lymphocyte count (%)
linfocitos_21786624_min	Minimum lymphocyte count (%)
linfocitos_21786624_ult_med_alt	Alteration in the latest measured median lymphocyte count (%)
linfocitos_21786624_ult_med_num	Number of measurements for the latest measured median lymphocyte count (%)
linfocitos_Âµl_21787060_media	Mean lymphocyte count (µL)
linfocitos_Âµl_21787060_mediana	Median lymphocyte count (µL)
linfocitos_Âµl_21787060_min	Minimum lymphocyte count (µL)
magnesio_meq_l_21786650_ult_med_alt	Alteration in the latest measured magnesium level (mEq/L)
max_horas_cirurgia	Maximum operative duration (in hours)
media_dd_temp_internacao_hist	Mean length of stay of previous admissions (in days)
mediana_dd_temp_internacao_hist	Median length of stay of previous admissions (in days)
metamielocitos_µl_21787820_ult_med_alt	Alteration in the latest measured metamyelocyte count (µL)
metamielocitos_21787852_ult_med_alt	Alteration in the latest measured metamyelocyte count
mieloblastos_µl_21785176_ult_med_alt	Alteration in the latest measured myeloblast count (µL)
mieloblastos_21786610_ult_med_alt	Alteration in the latest measured myeloblast count
mielocitos_µl_21785628_ult_med_alt	Alteration in the latest measured myelocyte count (µL)
mielocitos_21785964_alterado_relativo	Number of altered myelocyte count results relative to the reference value
mielocitos_21785964_total_exms	Total number of myelocyte count exams
mielocitos_21785964_total_positivos	Total number of positive results in myelocyte count
monocitos_µl_21787316_max	Maximum monocyte count (µL)
monocitos_µl_21787316_media	Mean monocyte count (µL)
monocitos_µl_21787316_mediana	Median monocyte count (µL)
monocitos_µl_21787316_min	Minimum monocyte count (µL)
monocitos_µl_21787316_ult_med_alt	Alteration in the latest measured mean monocyte count (µL)
monocitos_µl_21787316_ult_med_num	Number of measurements for the latest measured mean monocyte count (µL)
monocitos_21785128_alterado_relativo	Number of altered monocyte count results relative to the reference value
monocitos_21785128_diff_alt	Difference between the current measurement and the last altered monocyte count
monocitos_21785128_diff_num	Difference between the current measurement and the last numerical monocyte count
monocitos_21785128_freq_positivos	Frequency of positive results in monocyte count

continue...

...Continuation

Table 2S. List of covariates included in the study, including their names as shown in the dataset and a description detailing what each covariate refers to

Covariate name in the dataset	Description
monocitos_21785128_max	Maximum monocyte count
monocitos_21785128_media	Mean monocyte count
monocitos_21785128_mediana	Median monocyte count
monocitos_21785128_min	Minimum monocyte count
monocitos_21785128_std	Standard deviation of monocyte count
monocitos_21785128_total_exms	Total number of monocyte count exams
monocitos_21785128_total_positivos	Total number of positive results in monocyte count
monocitos_21785128_ult_med_alt	Last altered monocyte count measurement
monocitos_21785128_ult_med_num	Last numerical monocyte count measurement
monocitos_Âµl_21787316_media	Mean monocyte count (µL)
monocitos_Âµl_21787316_mediana	Median monocyte count (µL)
monocitos_Âµl_21787316_min	Minimum monocyte count (µL)
n_comorb_charlson	Number of comorbidities in Charlson Comorbidity Index
n_dias_uti	Number of days in ICU
n_reints_hist	Number of previous readmissions in the HIAE
neoplasia_maligna_secundaria_nao_especificada_linfonodos	Presence of secondary unspecified malignant neoplasm in lymph nodes
neoplasia_mama_orgaos_genitais_femininos	Presence of neoplasms in breast and female genital organs
neoplasia_orgaos_genitais_masculinos	Presence of neoplasms in male genital organs
neoplasia_ossos_articulacoes	Presence of neoplasms in bones and articulations
neoplasia_pele	Presence of skin neoplasms
neoplasia_sistema_respiratorio	Presence of neoplasms in the respiratory system
neoplasia_tecidos_mesoteliais_moles	Presence of neoplasms in mesothelial and soft tissues
neoplasia_trato_urinario	Presence of neoplasms in the urinary tract
neoplasias_glandulas_endocrinas	Presence of neoplasms in endocrine glands
neoplasias_malignas_tecidos_linfoides_hematopoieticos	Presence of malignant neoplasms in lymphoid and hematopoietic tissues
neoplasias_olho_cefalo_snc	Presence of neoplasms in eye, brain, and central nervous system
neoplasias_orgaos_digestivos	Presence of neoplasms in digestive organs
neutrofilo_parcial_none_92338688_alterado_relativo	Number of altered results of neutrophil partial count (unit unspecified) relative to the reference value
neutrofilo_parcial_none_92338688_total_exms	Total number of neutrophil partial count exams (unit unspecified)
neutrofilo_parcial_none_92338688_total_positivos	Total number of positive results in neutrophil partial count (unit unspecified)
neutrofilos_µl_21787308_ult_med_alt	Alteration in the latest measured neutrophil count (µL)
neutrofilos_21785780_media	Mean neutrophil count (%)
neutrofilos_21785780_mediana	Median neutrophil count (%)
neutrofilos_21785780_min	Minimum neutrophil count (%)
neutrofilos_21785780_ult_med_alt	Alteration in the latest measured neutrophil count
num_cirurgias	Number of surgeries
pad_max	Maximum diastolic blood pressure
pad_mediana	Median diastolic blood pressure
pad_medio	Mean diastolic blood pressure
pad_min	Minimum diastolic blood pressure
pas_max	Maximum systolic blood pressure
pas_mediana	Median systolic blood pressure
pas_medio	Mean systolic blood pressure
pas_min	Minimum systolic blood pressure
pco2_gasometria_venosa_mm_hg_21787352_ult_med_alt	Alteration in the latest measured partial pressure of carbon dioxide in venous blood gas analysis (mmHg)
peso_mediana	Median weight (in kg)
peso_medio	Mean weight (in kg)

continue...

...Continuation

Table 2S. List of covariates included in the study, including their names as shown in the dataset and a description detailing what each covariate refers to

Covariate name in the dataset	Description
peso_var	Variance of weight (in kg)
plaquetas_ul_µl_65470545_alterado_relativo	Number of altered platelet count (µL) results relative to the reference value
plaquetas_ul_µl_65470545_diff_alt	Difference between the current measurement and the last altered platelet count (µL)
plaquetas_ul_µl_65470545_diff_num	Difference between the current measurement and the last numerical platelet count (µL)
plaquetas_ul_µl_65470545_media	Mean platelet count (µL)
plaquetas_ul_µl_65470545_mediana	Median platelet count (µL)
plaquetas_ul_µl_65470545_min	Minimum platelet count (µL)
plaquetas_ul_µl_65470545_std	Standard deviation of platelet count (µL)
plaquetas_ul_µl_65470545_total_exms	Total number of platelet count exams (µL)
plaquetas_ul_µl_65470545_total_positivos	Total number of positive results in platelet count (µL)
plaquetas_ul_µl_65470545_ult_med_alt	Last altered platelet count (µL) measurement
plaquetas_ul_µl_65470545_ult_med_num	Last numerical platelet count (µL) measurement
plaquetas_x103_ul_21687766_max	Maximum platelet count (x10^3/µL)
plaquetas_x103_ul_21687766_media	Mean platelet count (x10^3/µL)
plaquetas_x103_ul_21687766_mediana	Median platelet count (x10^3/µL)
plaquetas_x103_ul_21687766_min	Minimum platelet count (x10^3/µL)
plaquetas_x103_ul_21687766_ult_med_alt	Alteration in the latest measured median platelet count (x10^3/µL)
plaquetas_x103_ul_21687766_ult_med_num	Number of measurements for the latest measured median platelet count (x10^3/µL)
po2_gasometria_venosa_mm_hg_21786636_ult_med_alt	Alteration in the latest measured partial pressure of oxygen in venous blood gas analysis (mmHg)
potassio_meq_l_21784990_max	Maximum potassium level (mEq/L)
potassio_meq_l_21784990_media	Mean potassium level (mEq/L)
potassio_meq_l_21784990_mediana	Median potassium level (mEq/L)
potassio_meq_l_21784990_min	Minimum potassium level (mEq/L)
potassio_meq_l_21784990_ult_med_alt	Alteration in the latest measured median potassium level (mEq/L)
potassio_meq_l_21784990_ult_med_num	Number of measurements for the latest measured median potassium level (mEq/L)
presc_cardiologia	Indicator for prescription of cardiology medications
presc_cardiologia to presc_radiologia	Number of specified orders or consultations
presc_cardiovascular	Indicator for prescription of cardiovascular medications
presc_cirurgia	Indicator for prescription of surgical procedures
presc_consultas	Indicator for prescription of medical consultations
presc_procedimentos	Indicator for prescription of medical procedures
presc_radiologia	Indicator for prescription of radiology procedures
press_art_max	Maximum arterial pressure
press_art_max_fixed	Maximum fixed arterial pressure
press_art_mediana	Median arterial pressure
press_art_medio	Mean arterial pressure
press_art_medio_fixed	Mean fixed arterial pressure
press_art_min	Minimum arterial pressure
press_art_min_fixed	Minimum fixed arterial pressure
promielocitos_µl_21785794_ult_med_alt	Alteration in the latest measured promyelocyte count (µL)
promielocitos_21785056_ult_med_alt	Alteration in the latest measured promyelocyte count
proteina_c_reativa_mg_l_21787786_ult_med_alt	Alteration in the latest measured C-reactive protein level (mg/L)
qtd_dd_desde_ult_intern	Number of days since the last hospitalization
qtd_emergencias_6m_lace	Number of emergency department visits in the last 6 months
qtd_internacoes_hist	Number of previous hospitalizations that lasted more than 24h
rdw_21687760_max	Maximum red cell distribution width (RDW)
rdw_21687760_media	Mean red cell distribution width (RDW)
rdw_21687760_mediana	Median red cell distribution width (RDW)

continue...

...Continuation

Table 2S. List of covariates included in the study, including their names as shown in the dataset and a description detailing what each covariate refers to

Covariate name in the dataset	Description
rdw_21687760_min	Minimum red cell distribution width (RDW)
rdw_21687760_ult_med_alt	Alteration in the latest measured red cell distribution width (RDW)
rdw_21687760_ult_med_num	Number of measurements for the latest measured red cell distribution width (RDW)
saturacao_da_hb_gasometria_venosa_21786114_ult_med_alt	Alteration in the latest measured hemoglobin oxygen saturation in venous blood gas analysis
segmentados_µl_21785954_ult_med_alt	Alteration in the latest measured segmented neutrophil count (µL)
segmentados_21787756_ult_med_alt	Alteration in the latest measured segmented neutrophil count
sexo	Sex
sexo_male_male	Gender indicator (1 for male, 0 for female)
sodio_meq_l_21787224_max	Maximum sodium level (mEq/L)
sodio_meq_l_21787224_media	Mean sodium level (mEq/L)
sodio_meq_l_21787224_mediana	Median sodium level (mEq/L)
sodio_meq_l_21787224_min	Minimum sodium level (mEq/L)
sodio_meq_l_21787224_ult_med_alt	Alteration in the latest measured median sodium level (mEq/L)
sodio_meq_l_21787224_ult_med_num	Number of measurements for the latest measured median sodium level (mEq/L)
sodio_tlr_poct_meq_l_25401723_ult_med_alt	Alteration in the latest measured sodium level by point-of-care testing (mEq/L)
spo2_max	Maximum oxygen saturation
spo2_mediana	Median oxygen saturation
spo2_medio	Mean oxygen saturation
spo2_min	Minimum oxygen saturation
temp_axilar_max	Maximum axillary temperature
temp_axilar_mediana	Median axillary temperature
temp_axilar_medio	Mean axillary temperature
temp_axilar_min	Minimum axillary temperature
temperatura_°c_21785398_ult_med_alt	Alteration in the latest measured temperature (°C)
tgo_u_l_21785858_ult_med_alt	Alteration in the latest measured glutamic-oxaloacetic transaminase (GOT) level (U/L)
tgp_u_l_21787128_ult_med_alt	Alteration in the latest measured glutamic-pyruvic transaminase (GPT) level (U/L)
tp_atividade_21786042_ult_med_alt	Alteration in the latest measured activated partial thromboplastin time (APTT)
ureia_mg_dl_21687780_media	Mean urea (mg/dL)
ureia_mg_dl_21687780_mediana	Median urea (mg/dL)
ureia_mg_dl_21687780_min	Minimum urea (mg/dL)
ureia_mg_dl_21687780_ult_med_alt	Alteration in the latest measured urea level (mg/dL)
ureia_mg_dl_21687780_ult_med_num	Number of measurements for the latest measured urea level (mg/dL)
vcm_fl_21687748_media	Mean volume of mean corpuscular volume (VCM) (fl)
vcm_fl_21687748_mediana	Median volume of mean corpuscular volume (VCM) (fl)
vcm_fl_21687748_min	Minimum volume of mean corpuscular volume (VCM) (fl)
vcm_fl_21687748_ult_med_alt	Alteration in the latest measured mean corpuscular volume (VCM) level (fL)
vcm_fl_21687748_ult_med_num	Number of measurements for the latest measured mean corpuscular volume (VCM) level (fL)
volume_medio_plaquetario_fl_21687774_max	Maximum mean platelet volume (MPV) level (fL)
volume_medio_plaquetario_fl_21687774_media	Mean platelet volume (MPV) level (fL)
volume_medio_plaquetario_fl_21687774_mediana	Median mean platelet volume (MPV) level (fL)
volume_medio_plaquetario_fl_21687774_min	Minimum mean platelet volume (MPV) level (fL)
volume_medio_plaquetario_fl_21687774_ult_med_alt	Alteration in the latest measured mean platelet volume (MPV) level (fL)
volume_medio_plaquetario_fl_21687774_ult_med_num	Number of measurements for the latest measured mean platelet volume (MPV) level (fL)
y_eritroblastos_automacao_29618927_max	Maximum automated erythroblast count
y_eritroblastos_automacao_29618927_media	Mean automated erythroblast count
y_eritroblastos_automacao_29618927_mediana	Median automated erythroblast count
y_eritroblastos_automacao_29618927_min	Minimum automated erythroblast count
y_eritroblastos_automacao_29618927_ult_med_alt	Alteration in the latest measured automated erythroblast count

continue...

...Continuation

Table 2S. List of covariates included in the study, including their names as shown in the dataset and a description detailing what each covariate refers to

Covariate name in the dataset	Description
y_eritroblastos_automacao_29618927_ult_med_num	Number of measurements for the latest measured automated erythroblast count
volume_medio_plaquetario_fl_21687774_std	Standard deviation of mean platelet volume (fl)
fosfatase_alcalina_u_l_21785844_alterado_relativo	Relative alteration in alkaline phosphatase (U/L)
sodio_meq_l_21787224_std	Standard deviation of sodium (mEq/L)
dias_internacao_lace	Days of hospitalization (LACE)
calc_mews_var	Calculation of MEWS variable
leucocitos_µl_21687720_std	Standard deviation of leukocytes (µL)
creatinina_mg_dl_21686440_std	Standard deviation of creatinine (mg/dL)
hematocrito_21687740_std	Standard deviation of hematocrit
dosagem_de_glicose_mg_dl_21787974_media	Mean glucose dosage (mg/dL)
determinacao_de_glicemia_capilar_mg_dl_25287943_media	Mean capillary blood glucose determination (mg/dL)
sodio_meq_l_21787224_total_positivos	Total positive results of sodium (mEq/L)
creatinina_mg_dl_21686440_diff_num	Number of differential creatinine measurements (mg/dL)
hcm_pg_21687752_std	Standard deviation of mean corpuscular hemoglobin (pg)
dosagem_de_glicose_mg_dl_21787974_alterado_relativo	Relative alteration in glucose dosage (mg/dL)
dosagem_de_glicose_mg_dl_21787974_std	Standard deviation of glucose dosage (mg/dL)
ureia_mg_dl_21687780_diff_num	Number of differential urea measurements (mg/dL)
vcm_fl_21687748_std	Standard deviation of mean corpuscular volume (fl)
neutrofilos_21785780_total_positivos	Total positive results of neutrophils
gama_gt_u_l_21786350_total_positivos	Total positive results of gamma-glutamyl transferase (U/L)
eosinofilos_21785258_diff_num	Number of differential eosinophils
pad_var	PAD variable
bilirrubina_indireta_mg_dl_21786074_total_positivos	Total positive results of indirect bilirubin (mg/dL)
promielocitos_21785056_alterado_relativo	Relative alteration in promyelocytes
basofilos_21785664_diff_num	Number of differential basophils
vcm_fl_21687748_diff_num	Number of differential mean corpuscular volume measurements (fl)
basofilos_21785664_media	Mean basophil count (%)
gama_gt_u_l_21786350_alterado_relativo	Relative alteration in gamma-glutamyl transferase (U/L)
volume_medio_plaquetario_fl_21687774_diff_alt_0_0	Zero-centered standardized difference of mean platelet volume (fl)
ureia_mg_dl_21687780_max	Maximum urea level (mg/dL)
plaquetas_x103_ul_21687766_std	Standard deviation of platelets (x10^3/µL)
ureia_mg_dl_21687780_total_positivos	Total positive results of urea (mg/dL)
tgp_u_l_21787128_alterado_relativo	Relative alteration in alanine aminotransferase (ALT) (U/L)
leucocitos_urina_ml_21785912_alterado_relativo	Relative alteration in leukocytes in urine (mL)
linfocitos_µl_21787060_diff_num	Number of differential lymphocytes (µL)
dosagem_de_glicose_mg_dl_21787974_diff_num	Number of differential glucose dosage measurements (mg/dL)
potassio_meq_l_21784990_std	Standard deviation of potassium (mEq/L)
ureia_mg_dl_21687780_alterado_relativo	Relative alteration in urea (mg/dL)
pas_var	PAS variable
chcm_g_dl_21687758_std	Standard deviation of mean corpuscular hemoglobin concentration (g/dL)
leucocitos_x103_ul_21687712_diff_num	Number of differential leukocytes (x10^3/µL)
linfocitos_21786624_diff_num	Number of differential lymphocytes
sexo_masculino	Male gender
determinacao_de_glicemia_capilar_mg_dl_25287943_diff_num	Number of differential capillary blood glucose determinations (mg/dL)
rdw_21687760_diff_num	Number of differential red blood cell distribution width measurements
monocitos_µl_21787316_diff_num	Number of differential monocytes (µL)
bilirrubina_direta_mg_dl_21785520_alterado_relativo	Relative alteration in direct bilirubin (mg/dL)
linfocitos_21786624_std	Standard deviation of lymphocytes

continue...

...Continuation

Table 2S. List of covariates included in the study, including their names as shown in the dataset and a description detailing what each covariate refers to

Covariate name in the dataset	Description
plaquetas_x103_ul_21687766_diff_num	Number of differential platelets (x10^3/µL)
proteina_c_reativa_mg_l_21787786_alterado_relativo	Relative alteration in C-reactive protein (mg/L)
sodio_meq_l_21787224_diff_num	Number of differential sodium measurements (mEq/L)
eosinofilos_21785258_std	Standard deviation of eosinophils
spo2_var	SpO2 variable
potassio_meq_l_21784990_diff_num	Number of differential potassium measurements (mEq/L)
ureia_mg_dl_21687780_std	Standard deviation of urea (mg/dL)
determinacao_de_glicemia_capilar_mg_dl_25287943_ult_med_num	Number of measurements for the latest median capillary blood glucose determination (mg/dL)
eosinofilos_µl_21787514_diff_num	Number of differential eosinophils (µL)
basofilos_21785664_max	Maximum basophil count (%)
basofilos_µl_21785724_alterado_relativo	Relative alteration in basophil count (µL)
press_art_var	Arterial pressure variable
monocitos_µl_21787316_std	Standard deviation of monocytes (µL)
temp_axilar_var	Axillary temperature variable
tgp_u_l_21787128_total_positivos	Total positive results of aspartate aminotransferase (AST) (U/L)
hemoglobina_g_dl_21687734_alterado_relativo	Relative alteration in hemoglobin (g/dL)
tgo_u_l_21785858_alterado_relativo	Relative alteration in glutamic-oxaloacetic transaminase (GOT) (U/L)
chcm_g_dl_21687758_diff_num	Number of differential mean corpuscular hemoglobin concentration measurements (g/dL)
creatinina_mg_dl_21686440_alterado_relativo	Relative alteration in creatinine (mg/dL)
basofilos_µl_21785724_diff_num	Number of differential basophil count measurements (µL)
hcm_pg_21687752_diff_num	Number of differential mean corpuscular hemoglobin measurements (pg)
sodio_meq_l_21787224_alterado_relativo	Relative alteration in sodium (mEq/L)
hematocrito_21687740_diff_num	Number of differential hematocrit measurements
determinacao_de_glicemia_capilar_mg_dl_25287943_std	Standard deviation of capillary blood glucose determination (mg/dL)
rdw_21687760_std	Standard deviation of red blood cell distribution width
volume_medio_plaquetario_fl_21687774_diff_num	Number of differential mean platelet volume measurements (fl)

Table 3S. Hyperparameters and performance metrics for each of the five different Machine Learning approaches tested to predict 30-day unplanned readmission using the “48-hour” model and the “discharge” model

Model	ML approach	Hyperparameters	AUC	PRAUC	Precision	F1	Fβ	True positive rate	True negative rate	False negative rate	False positive rate
48-hour	Logistic Regression	{‘C’: 0.019407941326836067, ‘max_iter’: 192, ‘class_weight’: {0: 1, 1: 15}, ‘n_jobs’: -1}	0.70 [0.68-0.72]	0.37 [0.35-0.41]	0.35 [0.31-0.38]	0.46 [0.43-0.48]	0.57 [0.54-0.60]	0.70 [0.61-0.74]	0.65 [0.61-0.71]	0.30 [0.26-0.39]	0.35 [0.29-0.39]
	Extra Tree	{‘n_estimators’: 96, ‘min_samples_split’: 91, ‘min_samples_leaf’: 9, ‘max_features’: 55, ‘class_weight’: {0: 1, 1: 8}, ‘max_depth’: 4, ‘max_samples’: 0.5545879975204516, ‘criterion’: ‘log_loss’, ‘bootstrap’: True, ‘oob_score’: False, ‘random_state’: 42, ‘n_jobs’: -1}	0.69 [0.67-0.71]	0.37 [0.34-0.40]	0.32 [0.31-0.36]	0.45 [0.42-0.46]	0.57 [0.53-0.60]	0.72 [0.62-0.77]	0.60 [0.53-0.68]	0.28 [0.23-0.38]	0.40 [0.32-0.47]
	Light GBM	{‘n_estimators’: 255, ‘min_samples_split’: 0.015403539023988118, ‘min_samples_leaf’: 0.014890111281431162, ‘bootstrap’: True, ‘max_features’: 100, ‘class_weight’: {0: 1, 1: 10}, ‘n_jobs’: -1, ‘random_state’: 42}	0.69 [0.67-0.71]	0.38 [0.34-0.41]	0.32 [0.30-0.37]	0.43 [0.42-0.46]	0.55 [0.52-0.58]	0.67 [0.61-0.74]	0.63 [0.56-0.67]	0.33 [0.26-0.39]	0.37 [0.33-0.44]
	Random Forest	{‘n_estimators’: 25, ‘max_depth’: 4, ‘min_samples_split’: 0.0461724789084436, ‘min_samples_leaf’: 0.014429846260993362, ‘bootstrap’: True, ‘max_features’: 15, ‘class_weight’: ‘balanced’, ‘criterion’: ‘gini’, ‘n_jobs’: -1, ‘ random_state’: 42}	0.70 [0.68-0.71]	0.38 [0.35-0.41]	0.33 [0.30-0.36]	0.44 [0.42-0.46]	0.56 [0.54-0.58]	0.70 [0.62-0.77]	0.64 [0.55-0.71]	0.30 [0.23-0.38]	0.36 [0.29-0.45]
	Hist Gradient Boosting	{‘learning_rate’: 0.014275845042010496, ‘l2_regularization’: 0.03936550026958983, ‘class_weight’: {0: 1, 1: 6}, ‘random_state’: 42}	0.66 [0.64-0.68]	0.35 [0.33-0.38]	0.33 [0.30-0.38]	0.42 [0.40-0.45]	0.51 [0.48-0.55]	0.59 [0.52-0.70]	0.67 [0.58-0.76]	0.41 [0.30-0.48]	0.33 [0.24-0.42]

continue...

...Continuation

Table 3S. Hyperparameters and performance metrics for each of the five different Machine Learning approaches tested to predict 30-day unplanned readmission using the "48-hour" model and the "discharge" model

Model	ML approach	Hyperparameters	AUC	PRAUC	Precision	F1	Fβ	True positive rate	True negative rate	False negative rate	False positive rate
Discharge	Logistic Regression	{'penalty': 'l1', 'solver': 'liblinear', 'C': 0.16877121440900922, 'max_iter': 55000, 'class_weight': 'balanced', 'random_state': 42, 'n_jobs': -1}	0.73 [0.71-0.74]	0.40 [0.38-0.45]	0.36 [0.34-0.39]	0.48 [0.46-0.50]	0.59 [0.57-0.62]	0.71 [0.67-0.75]	0.66 [0.62-0.72]	0.29 [0.25-0.33]	0.34 [0.28-0.38]
	Extra Tree	{'n_estimators': 127, 'min_samples_split': 34, 'min_samples_leaf': 31, 'max_features': 50, 'class_weight': None, 'max_depth': 7, 'max_samples': 0.44072936185207084, 'criterion': 'log_loss', 'bootstrap': True, 'oob_score': False, 'random_state': 42, 'n_jobs': -1}	0.71 [0.69-0.73]	0.40 [0.37-0.44]	0.34 [0.32-0.37]	0.47 [0.44-0.48]	0.59 [0.57-0.61]	0.73 [0.68-0.78]	0.61 [0.57-0.69]	0.27 [0.22-0.32]	0.39 [0.31-0.43]
	Light GBM	{'n_estimators': 300, 'learning_rate': 0.0013388425858958336, 'objective': 'binary', 'bagging_fraction': 0.7, 'bagging_freq': 4, 'max_depth': 5, 'max_bin': 100, 'num_leaves': 60, 'min_child_samples': 300, 'min_child_weight': 0.001844771171798263, 'min_gain_to_split': 0.006915947984771079, 'colsample_bytree': 0.29999999999999993, 'reg_alpha': 0.21477097559232294, 'reg_lambda': 0.013689319913766648, 'scale_pos_weight': 10.0, 'sigmoid': 1.9575319992396976, 'boost_from_average': False, 'boosting_type': 'dart', 'seed': 42}	0.70 [0.68-0.72]	0.37 [0.34-0.40]	0.33 [0.31-0.35]	0.45 [0.43-0.47]	0.59 [0.56-0.60]	0.74 [0.67-0.76]	0.61 [0.55-0.68]	0.26 [0.24-0.33]	0.39 [0.32-0.45]
	Random Forest	{'n_estimators': 50, 'max_depth': 6, 'min_samples_split': 0.028115726145031335, 'min_samples_leaf': 0.015438024373283283, 'bootstrap': True, 'max_features': 49, 'class_weight': None, 'n_jobs': -1, 'random_state': 25}	0.70 [0.69-0.73]	0.38 [0.36-0.42]	0.35 [0.31-0.37]	0.46 [0.43-0.47]	0.57 [0.54-0.60]	0.70 [0.61-0.76]	0.65 [0.58-0.70]	0.30 [0.24-0.39]	0.35 [0.30-0.42]
	Hist Gradient Boosting	{'learning_rate': 0.01, 'max_iter': 156, 'max_leaf_nodes': 9, 'min_samples_leaf': 50, 'class_weight': {0: 1, 1: 2}, 'random_state': 42}	0.71 [0.68-0.72]	0.38 [0.36-0.42]	0.34 [0.30-0.37]	0.45 [0.43-0.48]	0.57 [0.55-0.59]	0.68 [0.62-0.75]	0.64 [0.58-0.70]	0.32 [0.25-0.38]	0.36 [0.30-0.42]

AUC: area under the curve; PRAUC: area under the precision-recall curve; IQR: interquartile range.

Table 4S. Performance metrics evaluated in the independent holdout test set (n=361) after probability calibration, which improved the Brier score in both models, with a reduction from 0.21 to 0.16 for the 48-hour model and from 0.25 to 0.16 for the discharge model, indicating enhanced calibration and greater accuracy of the predicted probabilities

Metric	48-hour (Random Forest)	Discharge (LightGBM)
ROC AUC	0.72	0.72
PR AUC	0.40	0.39
Precision	0.35	0.41
F1	0.48	0.46
Fβ	0.61	0.50
True Positive Rate	0.75	0.53
True Negative Rate	0.60	0.79
False Negative Rate	0.25	0.47
False Positive Rate	0.40	0.21
Brier Score (calibrated)	0.16	0.16

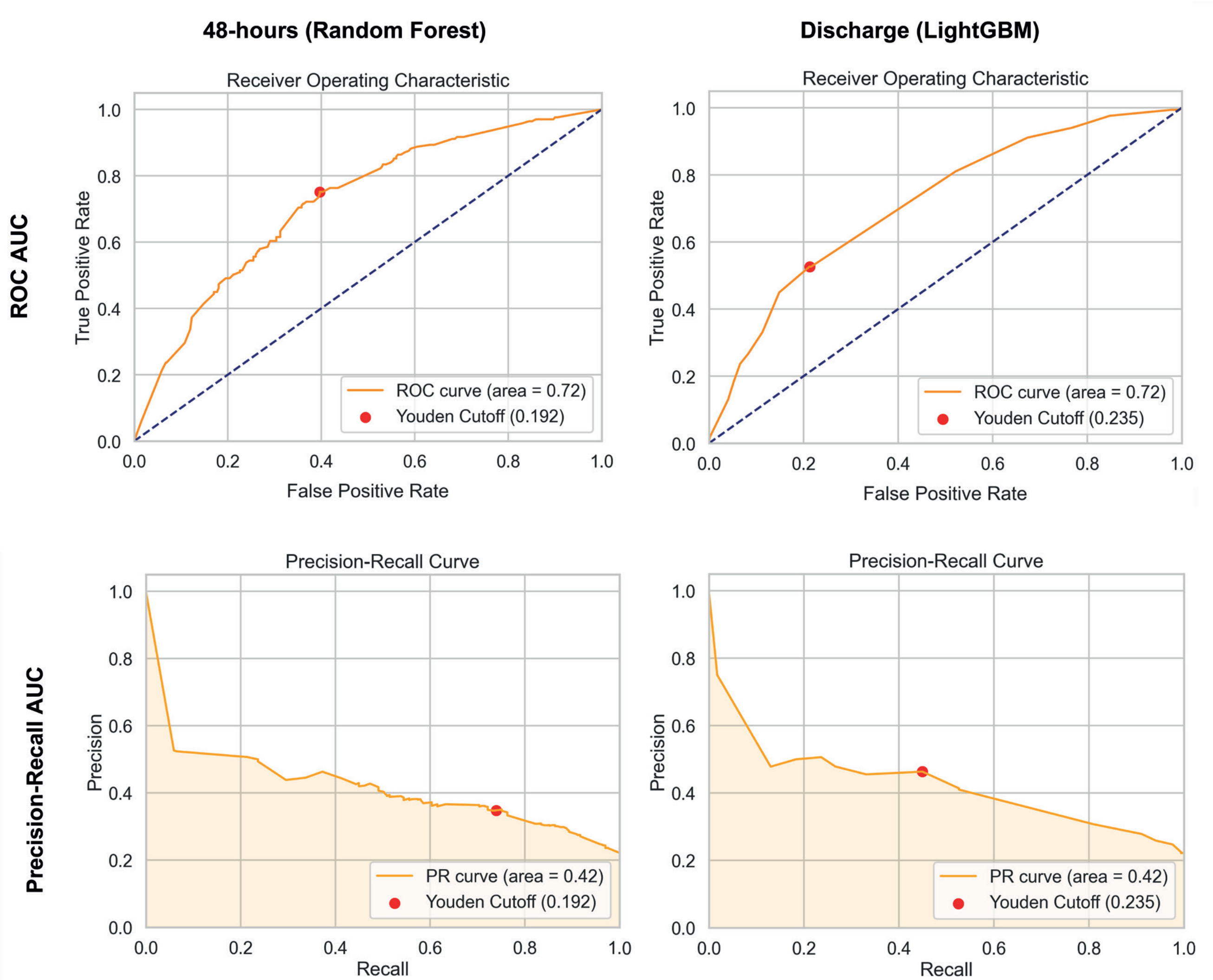

The red dot in each curve represents the Youden cutoff, which is the cutoff value for the predicted risk that maximizes sensitivity and specificity and can be used to dichotomize the prediction.

Figure 1S. Receiver operating characteristic and precision-recall curves from the test set for the Random Forest 48-hour model and LightGBM discharge model designed to predict 30-day unplanned readmissions in patients with cancer

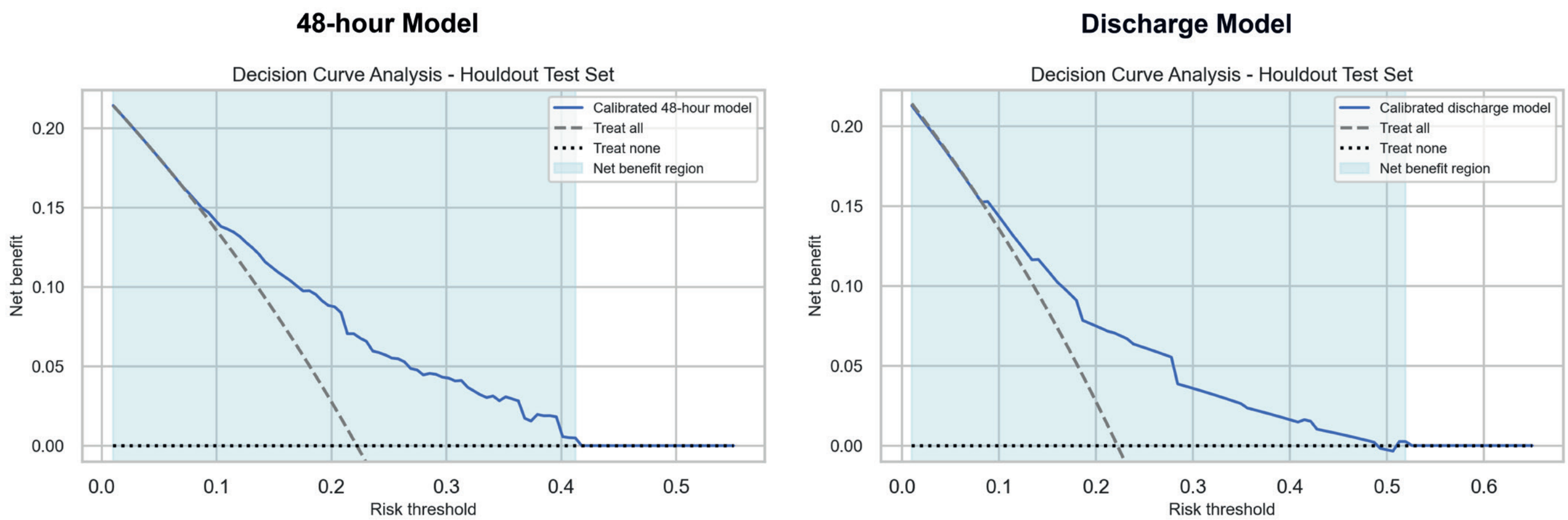

The Decision Curve Analysis (DCA) was performed to evaluate the clinical utility of the 48-hour and discharge models across a range of threshold probabilities. Both models demonstrated positive net benefit compared to the default strategies of "treat all" and "treat none" over a clinically relevant range of thresholds. Given the clinical goal of identifying patients at higher risk for unplanned readmissions, favoring higher sensitivity to avoid missing cases, the models were optimized for the Fβ-score (β=2), and the probability threshold was determined by the Youden method. These models provide greater net benefit, particularly in the lower to moderate threshold range (e.g., 10–25%), consistent with a screening strategy that accepts more false positives to minimize false negatives.

Figure 2S. Decision curve analysis comparing the net benefit of the 48-hour Random Forest model and the discharge LightGBM model for predicting 30-day unplanned readmissions
